# Biogas digestate as a sustainable phytosterol source for biotechnological cascade valorization

**DOI:** 10.1111/1751-7915.14174

**Published:** 2022-11-22

**Authors:** Tim Weckerle, Helen Ewald, Patrick Guth, Klaus‐Holger Knorr, Bodo Philipp, Johannes Holert

**Affiliations:** ^1^ Institute for Molecular Microbiology and Biotechnology Microbial Biotechnology & Ecology Group, University of Münster Münster Germany; ^2^ Institute of Landscape Ecology, Ecohydrology & Biogeochemistry Group University of Münster Münster Germany

## Abstract

Every year, several million tonnes of anaerobic digestate are produced worldwide as a by‐product of the biogas industry, most of which is applied as agricultural fertilizer. However, in the context of a circular bioeconomy, more sustainable uses of residual digestate biomass would be desirable. This study investigates the fate of the sterol lipids β‐sitosterol and cholesterol from the feedstocks to the final digestates of three agricultural and one biowaste biogas plants to assess if sterols are degraded during anaerobic digestion or if they remain in the digestate, which could provide a novel opportunity for digestate cascade valorization. Gas chromatographic analyses showed that feedstock sterols were not degraded during anaerobic digestion, resulting in their accumulation in the digestates to up to 0.15% of the dry weight. The highest concentrations of around 1440 mg β‐sitosterol and 185 mg cholesterol per kg dry weight were found in liquid digestate fractions, suggesting partial sterol solubilization. Methanogenic batch cultures spiked with β‐sitosterol, cholesterol, testosterone and β‐oestradiol confirmed that steroids persist during anaerobic digestion. *Mycobacterium neoaurum* was able to transform digestate sterols quantitatively into androstadienedione, a platform chemical for steroid hormones, without prior sterol extraction or purification. These results suggest that digestate from agricultural and municipal biowaste is an untapped resource for natural sterols for biotechnological applications, providing a new strategy for digestate cascade valorization beyond land application.

## Graphical abstract

In this study, we investigated the fate of the sterol lipids β‐sitosterol and cholesterol from the feedstocks to the final digestates of four biogas plants to assess if sterols are degraded during anaerobic digestion or if they remain in the digestate, which could provide a novel opportunity for digestate cascade valorization. We found that typical agricultural and biowaste feedstocks contain significant amounts of sterol lipids, which are not degraded during methanogenesis. Digestate from agricultural and municipal biowaste is therefore an untapped resource for natural sterol lipids, providing new possibilities for digestate cascade valorization beyond land application.

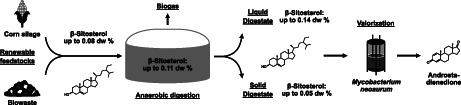

## INTRODUCTION

Biotechnological processes which convert waste streams of renewable organic material into valuable products provide great potential for the development of a sustainable circular (bio)economy. This applies in particular when the degree of biomass utilization is maximized through cascade valorization of the organic waste and all by‐ and end‐products generated during such processes (Keegan et al., 2013). In recent years, the biotechnological production of biogas from renewable substrates by anaerobic digestion has become an important practice in the development of a global circular (bio)economy. Biogas is the end‐product of a multistep biological process in which organic polymers and monomers, such as starch, cellulose and lipids from composite biomass, are transformed into methane (CH_4_) and carbon dioxide (CO_2_) by a complex microbial community in the absence of oxygen and other terminal electron acceptors (Hassa et al., 2018; Rudakiya & Narra, 2021; Weiland, 2010; Xu et al., 2021). Biogas use can help to mitigate climate change (Scarlat et al., 2018; Tilche & Galatola, 2008) by lowering the demand for fossil fuels either by on‐site production of electricity and heat or by further purification for use in gas grid distribution systems or as vehicle fuel (Monlau et al., 2015; Pucker et al., 2013).

However, the conversion of organic matter into biogas in anaerobic digestion is generally incomplete and produces significant amounts of residual solid and liquid digestate as a by‐product (Guilayn et al., 2020; Möller & Müller, 2012; Monlau et al., 2015). This digestate offers a great potential for cascade valorization because it contains high concentrations of undigested organic compounds, microbial biomass as well as inorganic components such as potassium, nitrogen and phosphorus compounds (Guilayn et al., 2022; Kaur et al., 2020; Monlau et al., 2015). Especially recalcitrant organic compounds like lignin and non‐hydrolysable lipids persist to a large extent or are only marginally transformed into smaller moieties and (pre‐)humic material under typical anaerobic digestor settings (Molinuevo‐Salces et al., 2013; Möller, 2015; Tambone et al., 2009). Driven by the continuous increase in the number of anaerobic digestor plants in the last decades, several hundred million tonnes of digestate are produced worldwide every year (Giwa et al., 2020; Kaur et al., 2020).

Despite its high potential for cascade valorization, anaerobic digestate is today predominantly used as organic fertilizer in agriculture and for soil improvement and amendment (Guilayn et al., 2022; Nkoa, 2014; Sheets et al., 2015; Tambone et al., 2010). However, this practice has raised serious concerns regarding its general sustainability because over‐fertilization, nutrient runoff, eutrophication of surface waters, spreading of pathogens and ammonium and nitrous oxide emission caused by intense digestate land application pose significant environmental risks on soils, water bodies and the atmosphere (Kaur et al., 2020; Monlau et al., 2015; Nkoa, 2014). Based on these concerns, restrictive regulations of digestate land application have been enacted by legislative bodies in most countries with significant biogas production capacities (European Parliament, 2019; U.S. Environmental Protection Agency, 2021; Wang et al., 2020). Accordingly, cascade valorization of anaerobic digestate is increasingly used to maximize biomass utilization and minimize its adverse effects (Guilayn et al., 2022; Kaur et al., 2020; Monlau et al., 2015; Tumaševičiūtė & Ignatavičius, 2019). Thermo‐chemical valorization, such as pyrolysis, combustion or hydrothermal carbonization, generates additional energy and value‐added products from whole or solid digestate in the form of heat, bio‐char, bio‐oils or syngas (Guilayn et al., 2022; Monlau et al., 2015; Sheets et al., 2015; Szulc et al., 2021). Liquid digestate fractions are typically valorized by nutrient recovery technologies such as ammonia stripping and struvite precipitation to produce concentrated fertilizer solutions (Macura et al., 2019; Tampio et al., 2016; Törnwall et al., 2017). Additionally, whole digestate or the liquid or solid fractions have been used as feedstocks for the cultivation of microalgae, yeasts or other microorganisms for the production of, e.g. biodiesel, bioplastic, biopesticides or bioactive enzymes (Kaur et al., 2020; Monlau et al., 2015). While most of these digestate‐cascading strategies make use of the inorganic or poly‐ and oligomeric organic fractions, little research effort has been put into the valorization of readily available monomeric organic components of anaerobic digestate. One example of such components is steroid‐like compounds, which have been shown to remain in the digestate after anaerobic digestion in significant amounts (Ahmad & Eskicioglu, 2019; Lu, Sun, et al., 2018; Lu, Xiao, et al., 2018; Tambone et al., 2010). Since neither bacteria nor archaea typically involved in the biogas process synthesize steroids de novo (Wei et al., 2016), these steroids are presumably introduced into the fermentation process with the plant‐ and animal‐based feedstocks, which can contain relevant amounts of phytosterols and cholesterol respectively (Piironen et al., 2003; Ryan et al., 2007; Tyagi et al., 2007). Although a range of denitrifying bacteria are able to fully degrade sterols under anoxic conditions (Ismail & Chiang, 2011; Warnke et al., 2017), it is not known whether sterol degradation can also occur under fermentative and methanogenic conditions, and detailed information about steroid concentrations and about the fate of steroids in the biogas process is still scarce.

**FIGURE 1:**
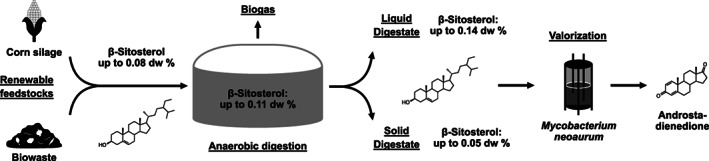
Schematic of a potential cascade valorization strategy for anaerobic digestate by using sterol lipids such as β‐sitosterol for biotechnological applications. β‐Sitosterol is introduced into the anaerobic digestion process with plant‐based feedstocks such as corn silage or biowaste. While other organic compounds are partially depleted and transformed into methane and carbon dioxide, sterols are not degraded under methanogenic biogas production conditions, leading to their stabilization in the final digestate. A proof‐of principle experiment showed that these sterols can be used directly for the biotechnological production of industrially important steroid intermediates, such as androstadienedione. β‐Sitosterol concentrations detected in this study are provided in % of the total dry weight (dw).

In this study, we investigated the fate of the sterol lipids β‐sitosterol (Figure 1) and cholesterol from the feedstocks to the final digestates of three agricultural and one biowaste‐treating biogas plants to assess whether sterols are degraded during anaerobic digestion or if they remain in the digestate. Sterols are in high demand for the biotechnological production of pharmaceutical steroid drugs, as food additives, for the cosmetics industry and as integral components of lipid nanoparticles (Batth et al., 2020; Donova, 2017; Feng et al., 2022; Holert et al., 2020; Hou et al., 2021), and their stabilization in anaerobic digestate could offer a novel opportunity for digestate cascade valorization.

## EXPERIMENTAL PROCEDURES

### Sample collection

Samples were collected from three agricultural biogas plants (plants 1–3) running wet fermentations and one dry fermentation biogas plant (plant 4) treating municipal biowaste (Table S1). Biogas plant 1 is located in the district of Warendorf (Münsterland region, Germany) and was operated continuously under mesophilic conditions (38–41°C). The plant consisted of one high‐load fermentation reactor and two serial post‐fermentation reactors and was equipped with a mechanical solid–liquid separator screw press. The main feedstocks in this plant were corn silage (ca. 80% w/w) and pig and cattle manure (ca. 20% w/w), along with minor amounts of rye and grass silage, and turkey manure. Samples from this plant were collected at five different time points (November 2019, January 2020, May 2020, January 2021 and April 2021; Table S1) and included the dry feedstock (corn silage), active methanogenic sludge from the first digestion tank and freshly separated solid and liquid digestate. At the January 2020 sampling, feedstock samples were not available and at the April 2021 sampling, feedstock and active methanogenic sludge samples were not available. Samples from the other three biogas plants were collected once. Biogas plant 2 is also located in the district of Warendorf and consisted of one main fermentation reactor and two serial post‐fermentation reactors and was run with corn silage (ca. 50% w/w) and pig and cattle manure (ca. 45% w/w) as main feedstocks at 37–38°C. Biogas plant 3 is located in the District of Coesfeld (Münsterland region, Germany) and consisted of one single fermentation reactor and was run with corn silage (ca. 40% w/w) and pig manure (ca. 60% w/w) with minor amounts of cattle manure as main feedstocks at 45–47°C. We collected corn silage feedstock samples and fresh digestate from these biogas plants (Table S1). The pig and cattle manure feedstocks of biogas plants 1–3 were fed directly into the first fermentation tanks and were not available for sampling. Biogas plant 4 uses biowaste collected in the Münsterland area (Germany) as feedstock in a dry fermentation process run at 34–38°C. We collected biowaste feedstock and fresh digestate of this biogas plant (Table S1). All liquid samples were filled into sterile glass bottles to the top to limit oxygen transfer into the samples and closed with screw caps. All dry samples were collected into sterile plastic tubes with screw caps. Samples were transferred to the laboratory within 1 h after sampling, and, if applicable, anaerobic batch cultures were inoculated with aliquots of the active methanogenic sludge samples immediately. All samples were prepared for steroid extraction directly (see below) or were stored at −20°C until further analysis. For sterol analysis, around 50 g wet weight of each sample was analysed. All raw data and calculations for these samples are summarized in Table S2.

### Anaerobic batch cultures

To investigate the fate of free sterols and other steroid compounds under methanogenic conditions, active methanogenic sludge was spiked with β‐sitosterol, cholesterol, testosterone and β‐oestradiol in three independent experiments. Spiked and unspiked control cultures were incubated under anoxic conditions, and steroid concentrations were measured over the course of up to 4 weeks. For each experiment, air‐tight 100‐ml serum bottles were autoclaved at 120°C for 35 min, before 0.05 millimoles of β‐sitosterol (Acros Organics, containing ca. 75% β‐sitosterol and 10% campesterol), cholesterol (AppliChem) or testosterone (Acros Organics, experiment 1), or a mix of testosterone and β‐oestradiol (Acros Organics, experiments 2 and 3) was added as solids. Serum bottles were inoculated by adding 50 ml aliquots of fresh methanogenic fermentation sludge collected from the main digestion tank of biogas plant 1 (Table S1) through a funnel while the headspace was continuously flushed with sterile nitrogen gas. The bottles were closed with butyl rubber septa, capped with aluminium closures and incubated at 37°C and 120 rpm. For steroid extractions, complete cultures were harvested immediately after inoculation and after 14 (experiments 1–3) and 28 days (experiments 2–3).

Production of biogas was monitored by daily measuring the changes in the cultures' headspace pressure and by determining CO_2_ and CH_4_ concentrations using gas chromatography on a weekly basis. Changes in the headspace pressure were determined by piercing the rubber septa with sterile cannulas attached to 20‐ml disposable syringes. The produced gas was released from the headspace into the syringe by releasing the plunger, and the resulting gas volumes were read from the syringe. In experiment 2, recording of biogas production was started after 3 days of incubation. The CO_2_ and CH_4_ concentrations in the produced biogas were measured by injecting 3 ml nitrogen gas into the headspace before injecting 3 ml of the resulting gas into an SRI 8610C gas chromatograph (GC; SRI Instruments) equipped with a methanizer and a flame ionization detector (FID). This was done before measuring the changes in the headspace pressure. CO_2_ and CH_4_ concentrations were calculated based on standard curves using CO_2_ and CH_4_ concentrations between 10% and 50% (v/v).

### Aerobic batch cultures

To investigate the applicability of anaerobic digestate as a source for phytosterol substrates for biotechnological applications, digestate samples from digestor plant 1 were tested as sterol feedstocks for *Mycobacterium neoaurum* DSM 2967, which is used in industrial scales for the transformation of phytosterols into androstadienedione (ADD) (Li et al., 2018). For this, digestate samples were shock frozen at −80°C, dried to completeness for several days in a Beta 1–16 lyophilizer (Christ) and homogenized using an impact grinder (coffee grinder TSM6A013B, 180 W; Bosch GmbH).

In the first experiment, around 2 g of dried and homogenized solid digestate collected in April 2021 were supplied as a sterol source in each culture. In the second experiment, around 0.8 g of dried and homogenized active sludge, liquid digestate or solid digestate collected in January 2021 were supplied in each culture. Digestate substrates were weighed into Erlenmeyer culture flasks and were suspended in 20 ml (experiment 1) or 10 ml (experiment 2) phosphate‐buffered (pH 7.2) minimal medium (containing 6.1 g l^−1^ K_2_HPO_4_, 2.1 g l^−1^ NaH_2_PO_4_ and 1.1 g l^−1^ NH_4_Cl). After autoclaving at 120°C for 30 min, the media were complemented with 0.37 g l^−1^ MgSO_4_ × 7 H_2_O, 0.0015 g l^−1^ CaCl_2_ × 2 H_2_O and trace element solution SL10 (Widdel et al., 1983). In the first experiment, aerobic batch cultures were prepared with and without 1% (w/v) methyl‐β‐cyclodextrin for sterol solubilization, which was added as a solid prior to autoclaving. In the second experiment, all cultures were prepared with 1% (w/v) methyl‐β‐cyclodextrin.

Starter cultures of *M. neoaurum* were grown in culture flasks in a complex medium containing 4 g l^−1^ glucose, 10 g l^−1^ yeast extract and 10 g l^−1^ malt extract (pH 7.2) at 30°C and 200 rpm. Cultures in the stationary phase were washed with the aforementioned minimal medium and diluted into the phytosterol transformation media to an optical density at 600 nm (OD_600_) of 1. Aerobic batch cultures were incubated at 30°C and 200 rpm. In the first experiment, 0.5 ml samples were taken regularly for up to 90 h and were extracted three times with 1 ml methyl‐tert‐butylester (MTBE)/methanol (10:3, v/v) for ADD identification and quantification. At each extraction step, the organic phases were removed after centrifugation at 12,000 *g* for 10 min and combined. The organic extracts were dried under a gentle nitrogen stream, and the remaining solids were resuspended in 1 ml chloroform. ADD was identified and quantified using GC‐FID analysis as described below. In the second experiment, sterol substrates and ADD were quantified by GC‐FID analysis as described below by harvesting the complete cultures directly after inoculation and after 48 h of incubation.

### Sample preparation and steroid extraction

Samples from original biogas plants and anaerobic and aerobic batch cultures were dried, homogenized and extracted to detect and quantify steroids. For this, each sample was frozen at −80°C and lyophilized in a Beta 1–16 lyophilizer (Christ) to complete dryness for several days. Dry samples were homogenized using an impact grinder (coffee grinder TSM6A013B, 180 W; Bosch GmbH) and stored at −20°C until analysis. Before 0.2–0.4 g of these homogenized samples was used for organic extraction, 75 μl or 200 μl of a 20 mM cholestane solution in chloroform was added as an internal standard, and the chloroform was evaporated under a gentle air stream. Dry samples spiked with cholestane were resuspended in 4 ml desalted water, and steroids were extracted with 5 ml MTBE/methanol (10:3, v/v), followed by two extractions with 2 ml MTBE/methanol (10:3, v/v). At each extraction step, the organic phases were removed after centrifugation at 4000 *g* for 10 min, combined and dried with MgSO_4_. After an additional centrifugation step, the organic extracts were dried under a gentle nitrogen stream and the remaining solids were resuspended in 1 or 2 ml chloroform. Organic extracts were stored at −20°C until further analysis.

### Analysis of steroid compounds

Steroids were analysed by GC‐FID analysis. One hundred microlitre aliquots of the organic extracts were evaporated under a gentle nitrogen stream, and the residues were resuspended in 50 μl pyridine (VWR International). Steroids were derivatized with 50 μl *N*,*O*‐bis(trimethylsilyl)‐trifluoroacetamide containing 1% trimethylchlorosilane (TCI), incubated for 20 min at 60°C and 40 min at room temperature. Samples were analysed on a Nexis GC‐2030 FID system (Shimadzu) equipped with a split injector and an SH‐RTx‐35 capillary column (320 μm × 30 m, 0.25 μm film thickness; Shimadzu, Kyoto, Japan). Helium (99.999%; Air Liquide) was used as the carrier gas with a flow rate of 23.4 ml min^−1^ and a split ratio of 10:1 with an injector temperature of 320°C. The oven temperature programme was 220°C for 2 min, increased to 320°C at 20°C min^−1^ and held for 8 min. The detector temperature was set to 320°C with a hydrogen flow of 32 ml min^−1^, an air flow of 200 ml min^−1^ and nitrogen as make‐up gas with a flow rate of 23.4 ml min^−1^.

Cholestane, cholesterol, β‐sitosterol, testosterone, β‐oestradiol and ADD peaks were identified by comparison to authentic standards based on retention times. Sterol and ADD concentrations were calculated using linear regressions of cholesterol, β‐sitosterol and ADD standard curves from 0.039 mM to 2.5 mM (each standard spiked with 1 mM cholestane). Sterol peaks in the analysed samples as well as the standard samples were normalized with the internal standard cholestane peak by dividing the respective peak areas.

To confirm the presence of β‐sitosterol and cholesterol and to identify sterol and steroid hormone transformation products, selected organic extracts were also analysed by GC‐mass spectrometry (MS) on an Agilent Technologies 7010B MS Triple Quad GC–MS (Agilent) equipped with a split injector and a DB‐5MS ultra inert column (30 m × 250 μm × 30 m, 0.25 μm film thickness; Agilent). Helium (99.999%; Air Liquide) was used as carrier gas with a flow rate of 2.25 ml min^−1^ with an injector temperature of 280°C. The oven temperature programme was 104°C for 2 min, increased to 290°C at 15°C min^−1^ and held for 15 min. Ions were generated in positive ionization mode (ESI) at 230°C and 70 eV. Individual mass spectra were analysed with the NIST database (National Institute of Standards and Technology).

Steroid concentrations were summarized and evaluated in RStudio (version 1.4.1717) using R version 4.1.0 with the rstatix (v = 0.7.0), ggpubr (v = 0.4.0) and ggplot2 (v = 3.3.5) packages, calculating the mean and standard deviation as well as the interquartile range (iqr).

## RESULTS

### Steroid content in anaerobic digestor samples

**FIGURE 2:**
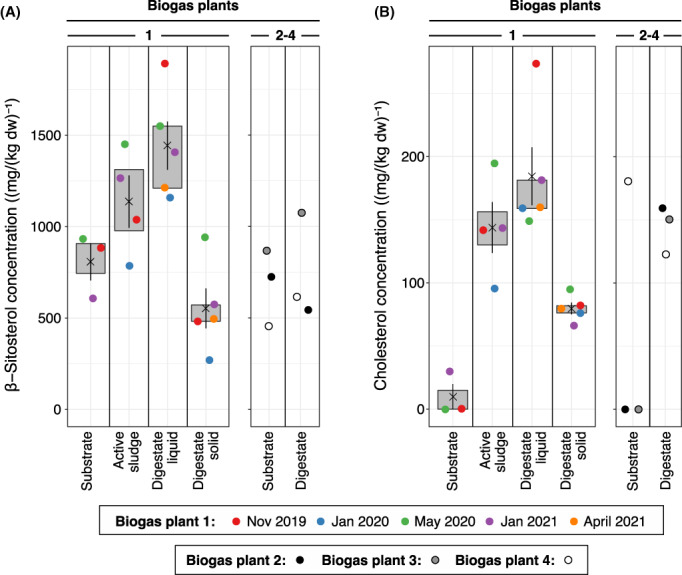
(A) β‐Sitosterol and (B) cholesterol content in samples from different stages of three agricultural anaerobic digestor plants (biogas plants 1–3) and one biogas plant treating municipal biowaste (biogas plant 4). Biogas plant 1 was sampled five times and average concentrations (black crosses), standard deviation (black lines) and interquartile ranges (grey boxes) are shown for these samples. β‐Sitosterol and cholesterol were extracted from freeze‐dried samples and quantified by GC‐FID analysis.

To determine the fate of sterols in biogas plants, we measured the concentrations of β‐sitosterol and cholesterol in different stages of three agricultural and one organic waste‐treating biogas plants. Agricultural biogas plant 1 was sampled five times over the course of 17 months and GC‐FID and GC–MS analysis showed that β‐sitosterol and cholesterol were present in all collected samples (Figure 2, Figures S1 and S2), except that cholesterol was not detected in two of three feedstock samples. Feedstock samples contained on average of 807 ± 175 mg β‐sitosterol per kilogram dry weight, while cholesterol was below the detection limit or present in minor amounts. Active sludge from the primary fermentation tank contained 1137 ± 287 mg (kg dw)^−1^ β‐sitosterol and 144 ± 40 mg (kg dw)^−1^ cholesterol. The liquid digestate contained 1443 ± 296 mg (kg dw)^−1^ β‐sitosterol and 185 ± 51 mg (kg dw)^−1^ cholesterol, while the solid digestate contained significantly lower amounts of both sterols with 553 ± 244 mg (kg dw)^−1^ β‐sitosterol and 80 ± 10 mg (kg dw)^−1^ cholesterol. Similar to biogas plant 1, the corn silage feedstock of the agricultural biogas plants 2 and 3 contained 723 mg (kg dw)^−1^ and 868 mg (kg dw)^−1^ β‐sitosterol, respectively, while cholesterol was not detected (Figure 2, Figure S1). Digestate from the final fermentation stage of biogas plants 2 and 3 contained 546 mg (kg dw)^−1^ and 1073 mg (kg dw)^−1^ β‐sitosterol and 159 mg (kg dw)^−1^ and 150 mg (kg dw)^−1^ cholesterol respectively. The biowaste feedstock from biogas plant 4 contained 457 mg (kg dw)^−1^ β‐sitosterol and 181 mg (kg dw)^−1^ cholesterol and the final digestate contained 614 mg (kg dw)^−1^ β‐sitosterol and 123 mg (kg dw)^−1^ cholesterol (Figure 2, Figure S1).

These analyses show that β‐sitosterol is present in significant amounts in corn silage and organic biowaste feedstocks and that the latter also contains significant amounts of cholesterol. In addition, the concentration of β‐sitosterol seems to increase slightly from the corn silage feedstock over the active methanogenic sludge to the liquid digestate in biogas plant 1. Altogether our data reveal that β‐sitosterol and cholesterol are not significantly degraded during anaerobic digestion.

### Methanogenic batch cultures with sterol additions

To confirm that sterols and other steroids are not degraded in active methanogenic communities, we spiked anoxic cultures that contained active biogas plant sludge as methanogenic biomass with β‐sitosterol, cholesterol or a mix of testosterone and β‐oestradiol. Steroids were quantified directly after inoculation and after 14 days (experiments 1–3) and 28 days of incubation (experiments 2–3).

**FIGURE 3:**
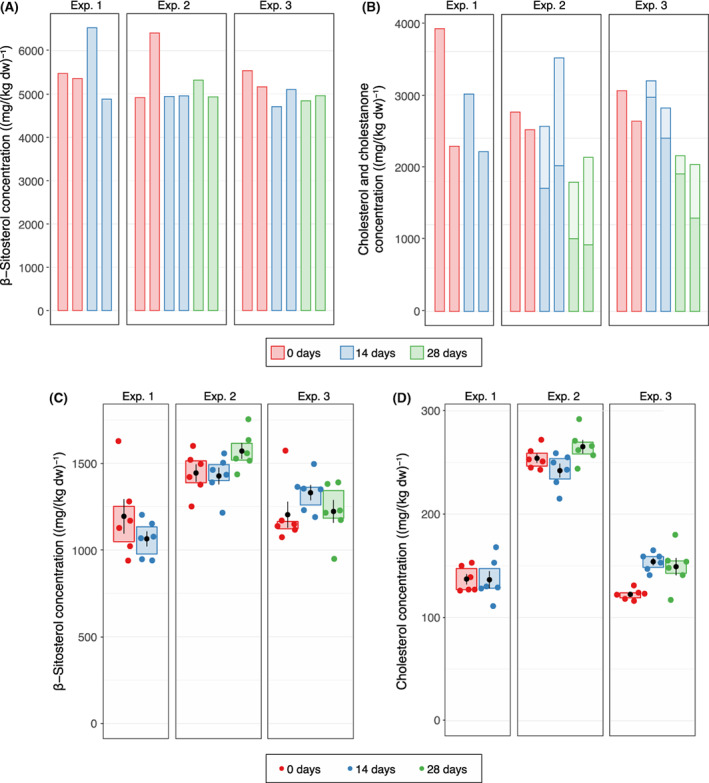
Sterol concentrations in methanogenic batch cultures from three independent experiments spiked with either β‐sitosterol or cholesterol and in cultures that were not spiked with the respective sterol substrate. (A) β‐Sitosterol concentrations in samples spiked with β‐sitosterol; (B) cholesterol (darker colour) and cholestane‐3‐one (lighter colour) concentrations in samples spiked with cholesterol; (C) β‐sitosterol concentrations in samples without added β‐sitosterol; (D) cholesterol concentrations in samples without added cholesterol. Whole cultures were harvested at the beginning of the experiment and after 14 and 28 days of incubation. β‐Sitosterol and cholesterol were extracted from freeze‐dried cultures and quantified by GC‐FID analysis. Black dots represent mean concentrations, black lines represent standard deviation and coloured boxes indicate the interquartile range.

The β‐sitosterol concentration in β‐sitosterol‐spiked samples remained stable over the whole incubation time in all three experiments (Figure 3A). The cholesterol concentration in cholesterol‐spiked samples remained stable until 14 days of incubation in all three experiments before it decreased by around 50%–60% after 28 days of incubation (Figure 3B). GC–MS analysis suggested that cholesterol was transformed into cholestan‐3‐one (Figure S3). Quantification of β‐sitosterol and cholesterol originating from the methanogenic inoculum in all samples that were not spiked with the respective sterol confirmed that the concentration of both sterols remained stable over the complete incubation time (Figure 3C,D).

Weekly measurement confirmed that all cultures continuously produced biogas with 36%–54% (v/v) CH_4_ and 33–50% (v/v) CO_2_ content throughout the complete incubation time (Figure S4). The amount of daily produced biogas declined in all cultures from around 30–50 ml at the beginning of the incubation to around 10–20 ml after 14 days (Figure S5). There was no significant difference in the total amount of produced biogas between the different samples, suggesting that the addition of steroids did not influence the biogas‐producing capability of the microbial community (Figure S5).

### Methanogenic batch cultures with steroid hormone additions

**FIGURE 4:**
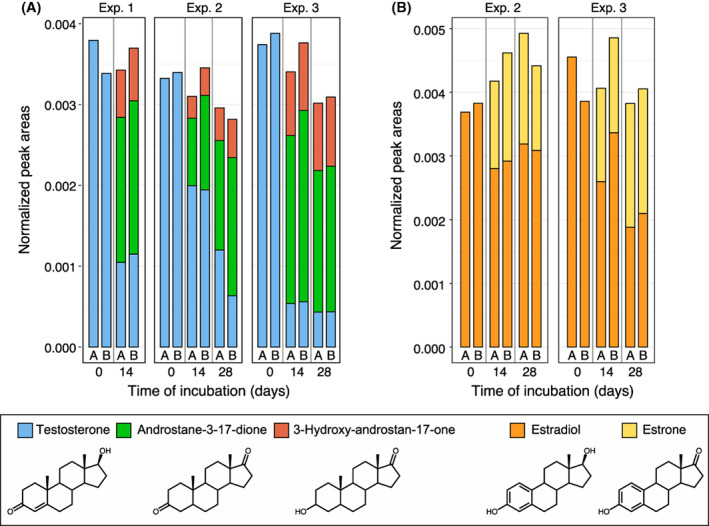
Steroid hormone concentrations in methanogenic batch cultures from three independent experiments spiked with (A) testosterone and (B) β‐oestradiol. Whole cultures from duplicate setups (A+B) were harvested at the beginning of the experiment and after 14 and 28 days of incubation. Testosterone, β‐oestradiol and their transformation products were extracted from freeze‐dried cultures and quantified by GC‐FID analysis.

Steroid hormones can be introduced into the anaerobic digestion process with manure feedstocks and the presence of steroid hormones in the final digestate can cause adverse effects in digestate land application (Rodriguez‐Navas et al., 2013). Although none of the digestate samples investigated in this study contained any detectable steroid hormones, we also investigated the fate of testosterone and β‐oestradiol under methanogenic conditions by spiking active methanogic cultures with a mix of these hormones. The concentration of testosterone decreased in all three experiments after 14 and 28 days of incubation to around 10%–30% of the initial concentration (Figure 4A). GC‐FID and GC–MS analyses revealed that testosterone was transformed into 3‐hydroxy‐androstan‐17‐one and androstane‐3‐17‐dione (Figure S6). Similarly, β‐oestradiol was partially oxidized to oestrone over time (Figure 4B, Figure S6). Overall, there was no significant net mass reduction in the tested steroid hormones and their transformation products.

### Aerobic *Mycobacterium neoaurum* batch cultures with anaerobic digestate

In the next step, we investigated whether anaerobic digestate is a potential substrate for the biotechnological production of steroid hormones by testing it as a sterol source for the androgen‐producing *Actinobacterium M. neoaurum*. To determine adequate incubation conditions, 20 ml cultures containing 2 g of freeze‐dried and homogenized solid digestate collected from biogas plant 1 in April 2020 were inoculated with *M. neoaurum* in the presence and absence of methyl‐β‐cyclodextrin. *M. neoaurum* produced around 0.25 mM androstadienedione (ADD) within 48 h in cultures with methyl‐β‐cyclodextrin (Figure S7) compared to a maximum of around 0.09 mM ADD in samples without methyl‐β‐cyclodextrin. This suggests that *M. neoaurum* can utilize sterols derived from the solid digestate in the presence of methyl‐β‐cyclodextrin to produce ADD.

**FIGURE 5:**
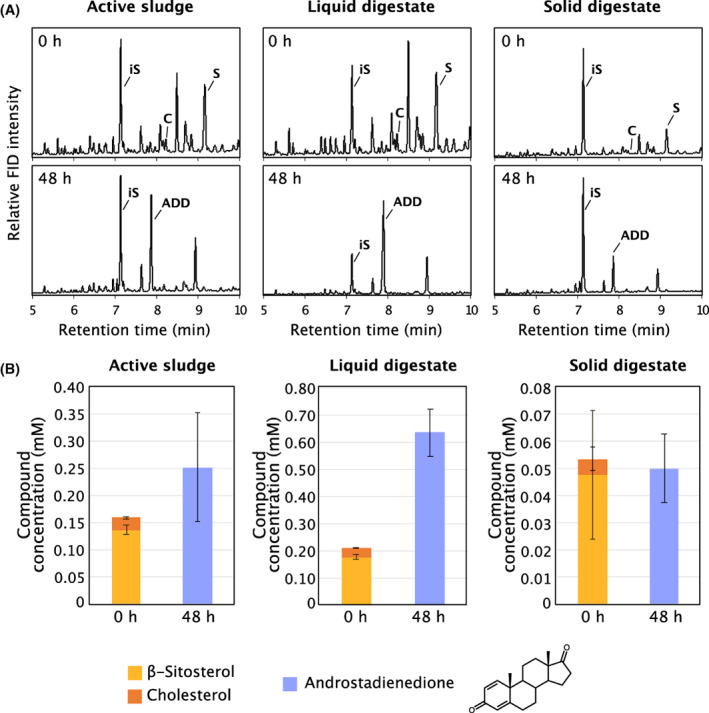
Formation of androstadienedione (ADD) from sterols in aerobic batch cultures of *Mycobacterium neoaurum* incubated with freeze‐dried and homogenized active methanogenic sludge, solid and liquid digestate from biogas plant 1. (A) Representative GC‐FID chromatograms of organic extracts from whole cultures harvested directly after inoculation (0 h) and after 48 h of incubation. iS = internal standard (cholestane); S = β‐sitosterol; C = cholesterol; ADD = androstadienedione. (B) Average concentrations of β‐sitosterol (yellow) and cholesterol (orange) after inoculation (0 h) and of ADD (blue) after 48 h of incubation. Black bars represent the standard deviation (*n* = 3).

Based on these results we set up a second experiment using active methanogenic sludge, liquid digestate and solid digestate as substrates for *M. neoaurum* in the presence of methyl‐β‐cyclodextrin. β‐Sitosterol and cholesterol were depleted from all cultures after 48 h of incubation and ADD accumulated as end‐product (Figure 5). Interestingly, *M. neoaurum* produced more ADD in cultures with active sludge and liquid digestate substrates than can be explained by the measured concentrations of β‐sitosterol and cholesterol in the cultures. This suggested that other sterol‐like compounds are present in the active sludge and the liquid digestate samples that can be transformed into ADD. Overall, these experiments confirmed that *M. neoaurum* can transform sterols that are present in the anaerobic digestate into ADD without the addition of any further substrates.

## DISCUSSION

Every year, several million tonnes of anaerobic digestate are produced globally as a by‐product of the growing biogas industry. Although anaerobic digestate is generally considered a valuable commodity predominantly used as fertilizer, the sheer volume of its accumulation and the ecological problems accompanying massive fertilization require the implementation of more sustainable and economical utilization routes. For this, anaerobic digestate needs to be established as a resource for additional cascade valorization to facilitate comprehensive biomass utilization throughout the whole biogas production process (Guilayn et al., 2022; Kaur et al., 2020; Monlau et al., 2015; Tumaševičiūtė & Ignatavičius, 2019). In this study, we show that valuable sterol lipids are not degraded during anaerobic digestion in typical agricultural and biowaste biogas plants and therefore remain in the final digestates, which provides a novel opportunity for digestate valorization as a sterol resource for biotechnological applications. In line with this, we demonstrate in a proof‐of‐principle experiment that an industrially used *Mycobacterium neoaurum* strain can be used to quantitatively transform digestate sterols into valuable steroid precursors for the pharmaceutical industry without prior sterol extraction (Figure 1).

Our data reveal that the sterol model compounds β‐sitosterol and cholesterol are present in significant amounts in typical biogas feedstocks and that both sterols are not degraded under the methanogenic conditions of anaerobic digestion. This is consistent with results from a previous study that showed that phytosterol degradation, although thermodynamically feasible, did not occur in long‐term semi‐continuously phytosterol‐fed methanogenic cultures inoculated with microbes from a pulp and paper wastewater treatment plant (Dykstra et al., 2014). In general, sterols can be fully degraded to CO_2_ and biomass by selected bacteria under oxic or denitrifying conditions (Chiang et al., 2008; Galan et al., 2019; Olivera & Luengo, 2019; Warnke et al., 2017; Wei et al., 2018). However, genes coding for an O_2_‐dependent sterol degradation pathway were found to be largely absent in anaerobic bioreactors (Bergstrand et al., 2016; Holert et al., 2018), suggesting that aerobic sterol degradation is prevented by the very low O_2_ concentrations (Botheju, 2011). Similar to O_2_, nitrate and nitrite concentrations are typically very low in anaerobic digestors, suggesting that the growth and sterol degradation activity of denitrifying sterol‐degrading bacteria are also repressed under methanogenic conditions. Although we observed the transformation of spiked cholesterol to cholestanone in our methanogenic batch cultures, there appeared to be only a marginal net mass reduction in these two sterols. Cholesterol can be reduced to coprostanol during anaerobic digestion (Ahmad & Eskicioglu, 2019) and cholestanone is an intermediate of this microbial process under anoxic conditions (Kenny et al., 2020). However, it remains unclear why the final oxidation step from cholestanone to cholestanol did not occur in our laboratory cultures.

We detected up to 0.8% β‐sitosterol (w/w) in the corn silage feedstocks of our investigated agricultural biogas plants, which is well in the range 0.2%–2% β‐sitosterol (w/w) that are typically found in different parts of the corn plant (Bacchetti et al., 2011; Harrabi et al., 2008; Ryan et al., 2007; Zhang et al., 2017). Conversely, we detected no or only small amounts of cholesterol in the plant‐based feedstock fraction which is consistent with the minor role that cholesterol plays in the sterol pool of most plants (Behrman & Gopalan, 2005; Sonawane et al., 2017). However, we detected up to 0.2% cholesterol in the active methanogenic sludge and the digestate of the investigated agricultural biogas plants, which was presumably introduced by the 20%–60% pig and cattle manure feedstocks (Tyagi et al., 2007). Unfortunately, the manure‐based feedstocks in our study were not available for sampling, which prevented an exact quantification of the amount of cholesterol and β‐sitosterol that was contributed by these feedstocks.

Our data further indicate that the β‐sitosterol concentration increased in some biogas plants during the methanogenic process similar to what has been reported for other recalcitrant organic compounds like lignin and non‐hydrolysable lipids (Möller, 2015; Tambone et al., 2009). This agrees with the notion that sterols are not degraded by the methanogenic community during the biogas process, while the total organic carbon concomitantly decreases due to a release of carbon dioxide and methane from fermentable substrates, which are depleted during biogas production. Strikingly, cholesterol and β‐sitosterol concentrations were higher in the liquid digestate fractions than in the solid fractions of biogas plant 1, suggesting that both sterols were partially solubilized during the fermentation process. While cholesterol is typically emulsified in soluble particles in animal faeces (Grefhorst et al., 2019), most β‐sitosterol is presumably embedded in the solid plant‐based feedstocks from which it is apparently released during the biogas process.

Natural sterols are in high demand for the production of pharmaceutically active steroid drugs, as food additives, for the cosmetics industry and as integral components of the growing market for lipid nanoparticles for drug or mRNA vaccine delivery (Batth et al., 2020; Donova, 2017; Feng et al., 2022; Fernandes & Cabral, 2007; Hou et al., 2021). Accordingly, the global demand for sterols has increased significantly in recent years (Borowitzka, 2013; Randhir et al., 2020) and anaerobic digestate could contribute a significant share of this market as a novel and sustainable source of natural sterols. Today, phytosterols are mainly extracted from vegetable oil and tall oil which typically contain 0.1%–10% phytosterols based on dry weight (Bacchetti et al., 2011; Fernandes & Cabral, 2007; Gonzalez‐Diaz et al., 2021). These concentrations are similar to the β‐sitosterol content we detected in anaerobic digestate, suggesting that the large quantities of agricultural biogas digestate represent a feasible phytosterol source for industrial applications. However, with respect to the food‐versus‐fuel dilemma of using energy crops as biogas plant feedstocks, a long‐term global transition to more sustainable organic waste stream feedstocks, such as food waste and municipal biowaste, is desirable. Our data reveal that the final digestate of a municipal biowaste‐treating biogas plant contained similar concentrations of β‐sitosterol and cholesterol as the agricultural biogas digestate, suggesting that further valorization of biowaste digestate as a sterol source is also conceivable. Industry‐scale technologies for the extractions and purification of lipids from complex matrices are already established, including organic extractions using green solvents (Byrne et al., 2016) and supercritical fluid extractions (Dikshit et al., 2020; Fernandes & Cabral, 2007). These methods could be adapted to the large‐scale extraction of sterols and other valuable lipids, such as archaeal ether lipids, from anaerobic digestate. In light of an increasing global sterol demand and a decreasing supply of sterols from slaughterhouse waste and the paper industry, the extraction of sterols from anaerobic digestate from renewable waste stream substrates could offer an economically feasible alternative. However, due to the complexity of the digestate substrate, further research into cost‐effective and environmentally friendly separation and purification techniques for these lipid classes are required to meet the strict standards of the pharmaceutical or cosmetics industries.

Our proof‐of principle experiment showed that *Mycobacterium neoaurum* was able to quantitatively transform digestate sterols into ADD, which is a platform chemical for the biotechnological production of steroid hormones (Donova, 2017), without previous sterol extraction or purification. Interestingly, *M. neoaurum* produced more ADD than what could be explained by the amount of β‐sitosterol and cholesterol in the digestate, suggesting that other sterol‐like compounds, such as bile acids, might be present in significant amounts in the digestate samples. For maximal ADD production, *M. neoaurum* required the solubilizing agent methyl‐β‐cyclodextrin, which is known to increase phytosterol transformations in *Actinobacteria* (Su et al., 2020). Alternative two‐phase fermentation techniques, which have been successfully used for the production of ADD from phytosterols in *Mycobacteria* (Shi et al., 2019; Xu et al., 2014), could provide a more sustainable way for ADD production from digestate phytosterols.

Besides the potential advantages of digestate valorization, high digestate steroid concentrations can also cause negative effects in digestate land applications. Although we did not detect any free steroid hormones in the biogas plant samples, pig and cattle manure feedstocks often contain androgenic and estrogenic steroid hormones (Hanselman et al., 2003; Hansen et al., 2011). Similar to other studies (de Mes et al., 2008; Muller et al., 2010), we found that spiked testosterone and β‐oestradiol were not depleted under methanogenic conditions, potentially leading to the accumulation of steroid hormones in biogas digestate (Rodriguez‐Navas et al., 2013). Steroid hormones pose significant risks if digestate is applied to agricultural land due to their endocrine activity (Stasinakis, 2012). High phytosterol concentrations in the digestate could also cause adverse effects after land application because phytosterols and similar compounds can act as substrates for aerobic steroid‐degrading bacteria, which can lead to the formation and accumulation of endocrine‐disrupting steroid hormones in the environment (Denton et al., 1985; Gravert et al., 2021; Jenkins et al., 2003, 2004; Mendelski et al., 2019). This potential ecological risk is also underlined by our transformation experiment with *M. neoaurum*, confirming that digestate phytosterol can in principle be transformed into endocrine compounds by microbes.

Taken together, anaerobic digestate from agricultural and municipal biowaste represents a distinguished bioresource for natural sterol lipids, exhibiting higher concentrations of solubilized sterols than the respective feedstocks, especially in liquid digestate fractions. Thus, sterol utilization for biotechnological and other industrial applications provides a great potential for cascade valorization of digestate, extending the options for digestate use beyond land application. In addition, the removal of phytosterols from digestate could help to reduce the endocrine effects of digestate when applied to agricultural soils. The remaining sterol‐depleted digestate residues can still be used for thermochemical valorization processes to generate additional energy or as feedstocks for the production of other value‐added products by microbial fermentations.

## AUTHOR CONTRIBUTIONS

**Tim Weckerle:** Formal analysis (equal); investigation (equal). **Helen Ewald:** Formal analysis (equal); investigation (equal). **Patrick Guth:** Resources (supporting). **Klaus‐Holger Knorr:** Resources (supporting); writing – original draft (supporting). **Bodo Philipp:** Conceptualization (equal); project administration (equal); supervision (equal); writing – original draft (supporting); writing – review and editing (supporting). **Johannes Holert:** Conceptualization (lead); data curation (lead); investigation (equal); methodology (equal); project administration (equal); supervision (equal); visualization (lead); writing – original draft (lead); writing – review and editing (lead).

## FUNDING INFORMATION

No funding information provided.

## CONFLICT OF INTEREST

The authors declare no conflict of interest.

## Supporting information


Appendix S1:

## Data Availability

All required experimental data of this study are contained within the manuscript. Data that support the findings of this study are available in the supplementary material of this article.
