# Supplementary material for: Biogas digestate as a sustainable phytosterol source for biotechnological cascade valorization
Source: Microb Biotechnol. 2022 Nov 22;16(2):337–49. doi: 10.1111/1751-7915.14174 (PMC9871531; doi:10.1111/1751-7915.14174)
Supplement: Supplementary file 1 — Appendix S1: [file MBT2-16-337-s001.zip › mbt214174-sup-0001-Supplemental_figures.pdf]

Supplementary material

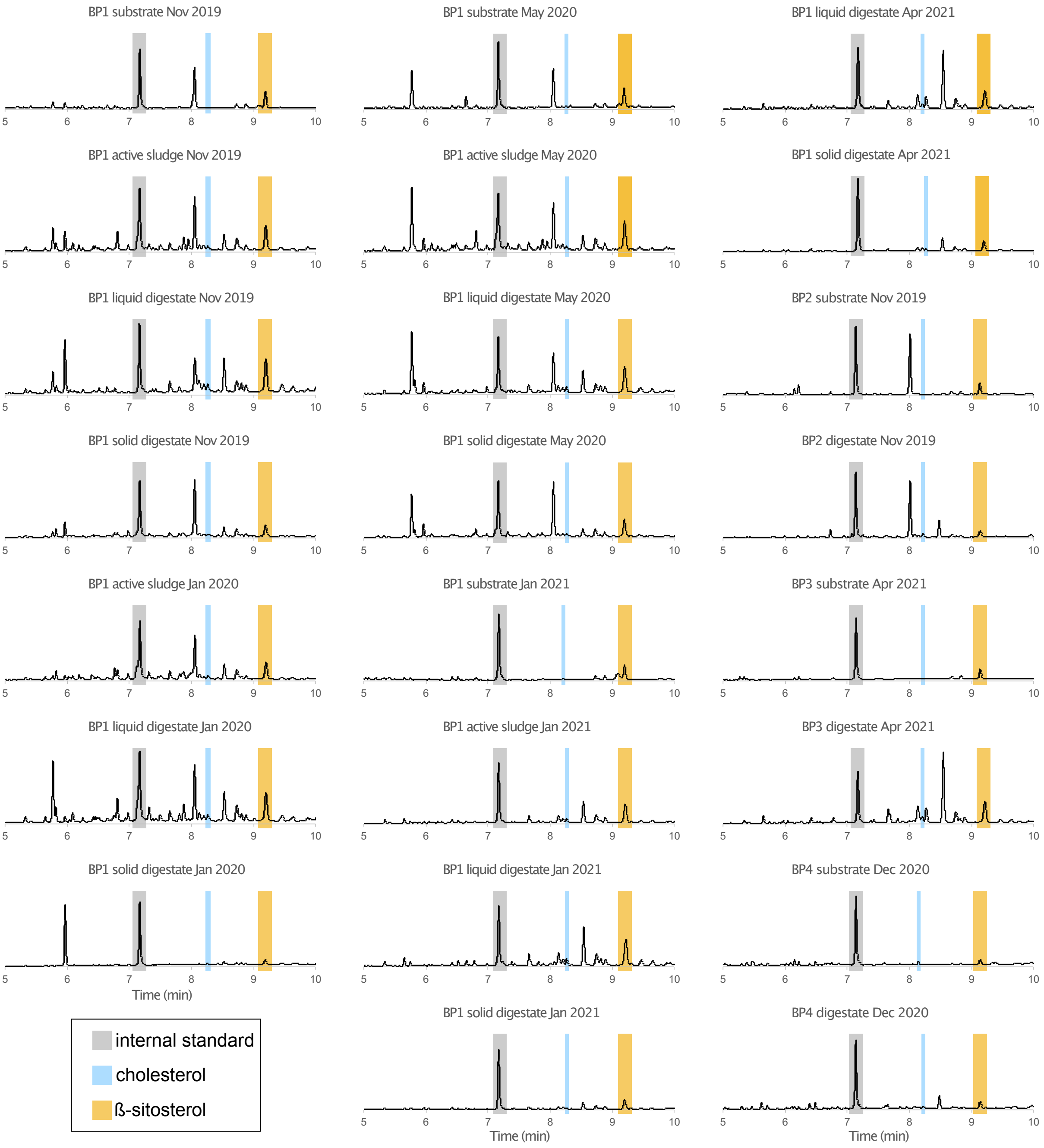

Supplemental figure S1: GC-FID chromatograms of organic lipid extracts from samples collected at different stages of four biogas plants (BP1-4). Internal standard (cholestane), cholesterol and β-sitosterol peaks are labelled with grey, blue and orange boxes, respectively.

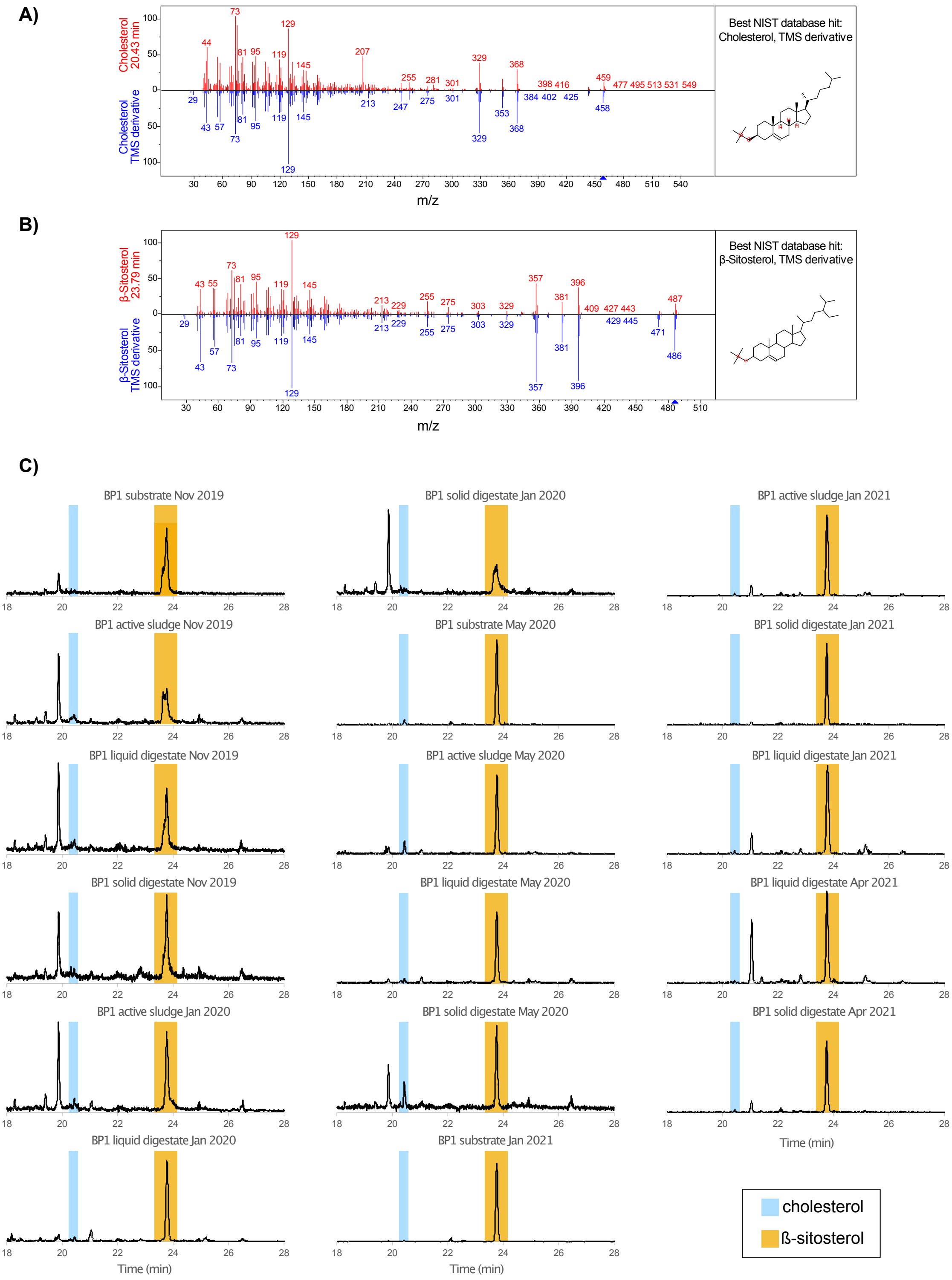

Supplemental figure S2: GC-MS analysis of lipid extracts from biogas plant 1. Ions were generated in positive ion mode. **(A)** Representative mass spectrum of a peak with the retention time of 20.43 minutes (red lines) and comparison to the mass spectrum of the best hit in the NIST database (blue lines). This peak was identified as the trimethylsilyl (TMS)-derivative of cholesterol. **(B)** Representative mass spectrum of a peak with the retention time of 23.79 minutes (red lines) and comparison to the mass spectrum of the best hit in the NIST database (blue lines). This peak was identified as the TMS-derivative of β-sitosterol. **(C)** Extracted ion chromatograms of samples collected at five different time points from biogas plant 1. The two major ions of cholesterol with m/z values of 368 and 458 and of β-sitosterol with m/z values of 396 and 486 were extracted for analysis. Cholesterol and β-sitosterol peaks are labelled with blue and orange boxes, respectively.

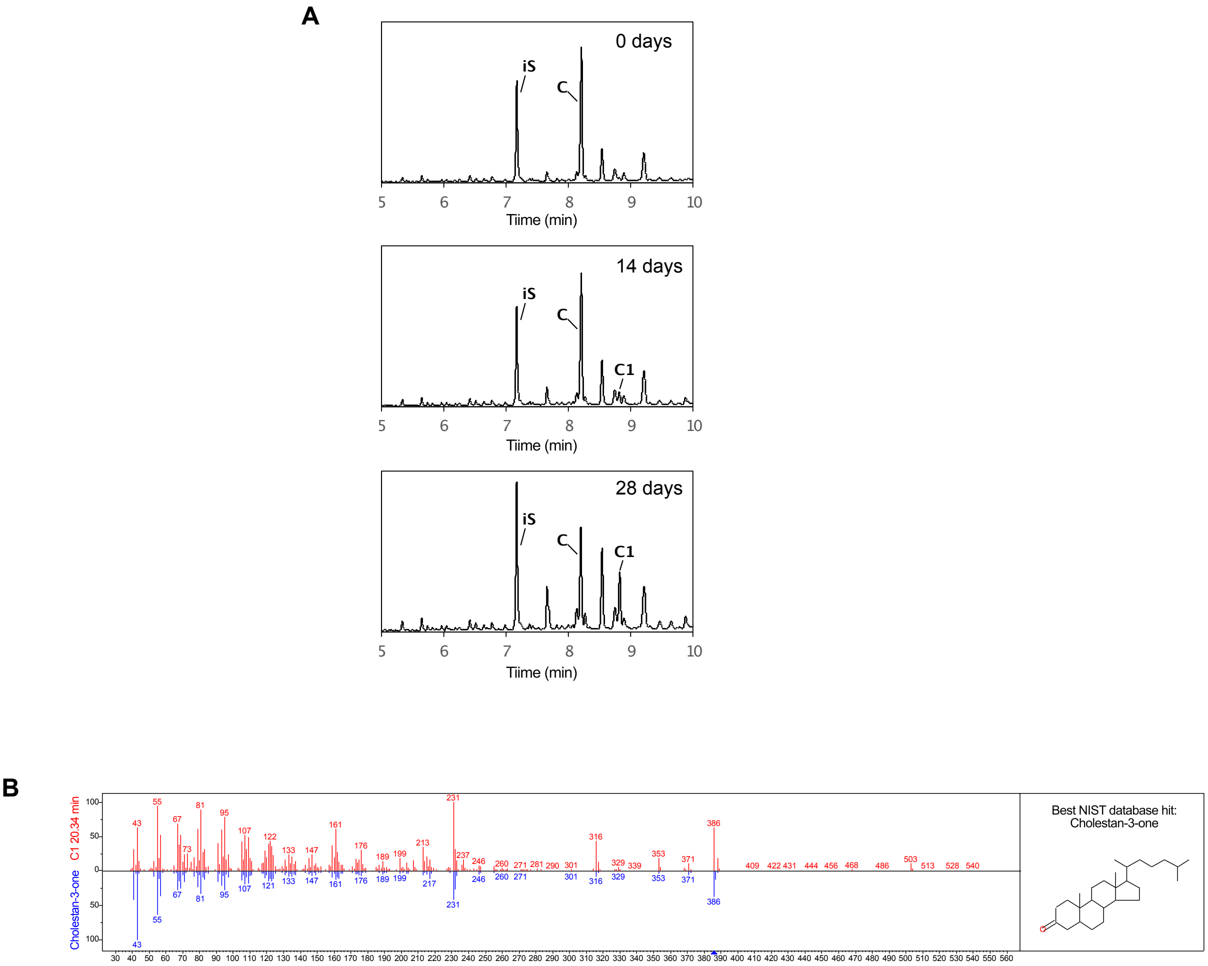

Supplemental figure S3: **(A)** Representative GC-FID chromatograms (experiment 3B) of organic lipid extracts from methanogenic batch cultures from three independent experiments spiked with cholesterol **(C)**. Whole cultures were harvested at the beginning of each experiment and after 14 and 28 days of incubation. Cholestane was used as internal extraction standard (**iS**). Cholesterol was partially transformed into a new product that was identified as **(B)** cholestan-3-one (**C1**) by GC-MS analysis.

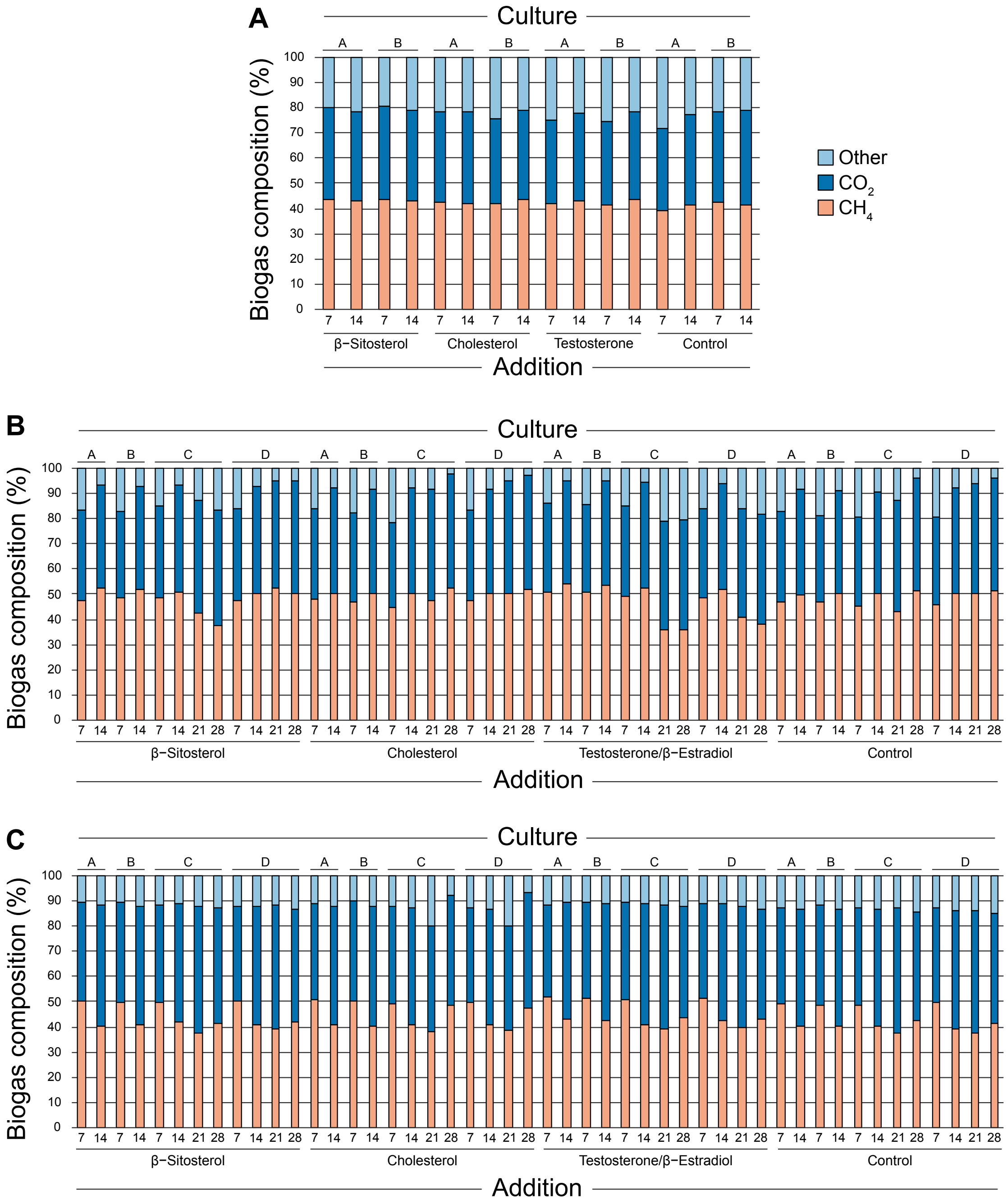

Supplemental figure S4: Biogas composition in methanogenic batch cultures spiked with different steroids and in control cultures without added steroids in three independent experiments **(A-C)**. Methane and carbon dioxide concentrations were determined by GC-FID analysis in all samples on a weekly basis. Cultures were harvested after 14 or 28 days

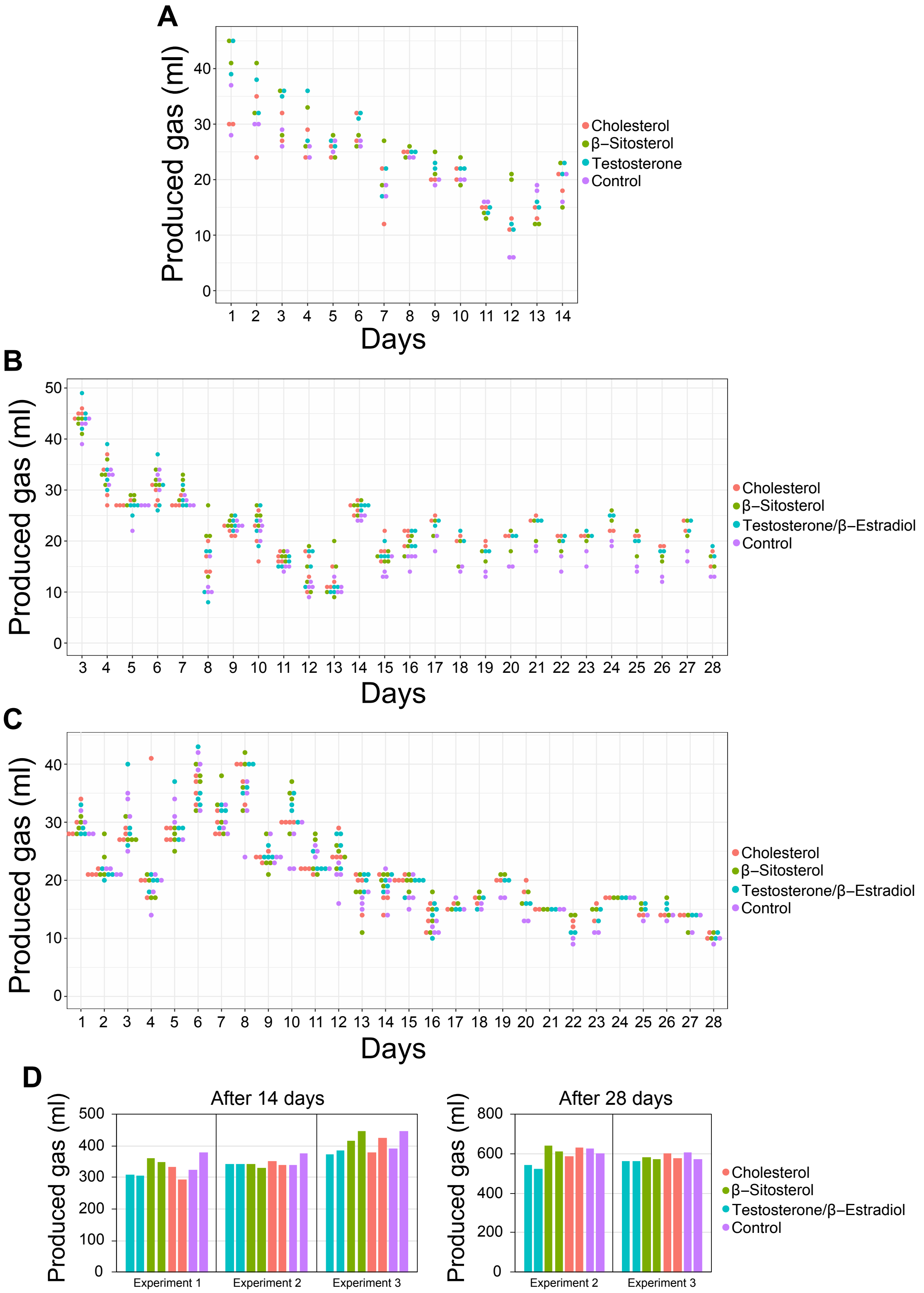

Supplemental figure S5: Daily changes in the headspace pressure of methanogenic batch cultures spiked with different steroids and in control cultures without added steroids in three independent **experiments expressed as volume of produced gas. (A-C)**. Produced gas volumes were determined by piercing the culture rubber septa with sterile cannulas attached to 20 ml syringes. **(D)** Total amount of produced gas after 14 and 28 days of incubation in all three experiments.

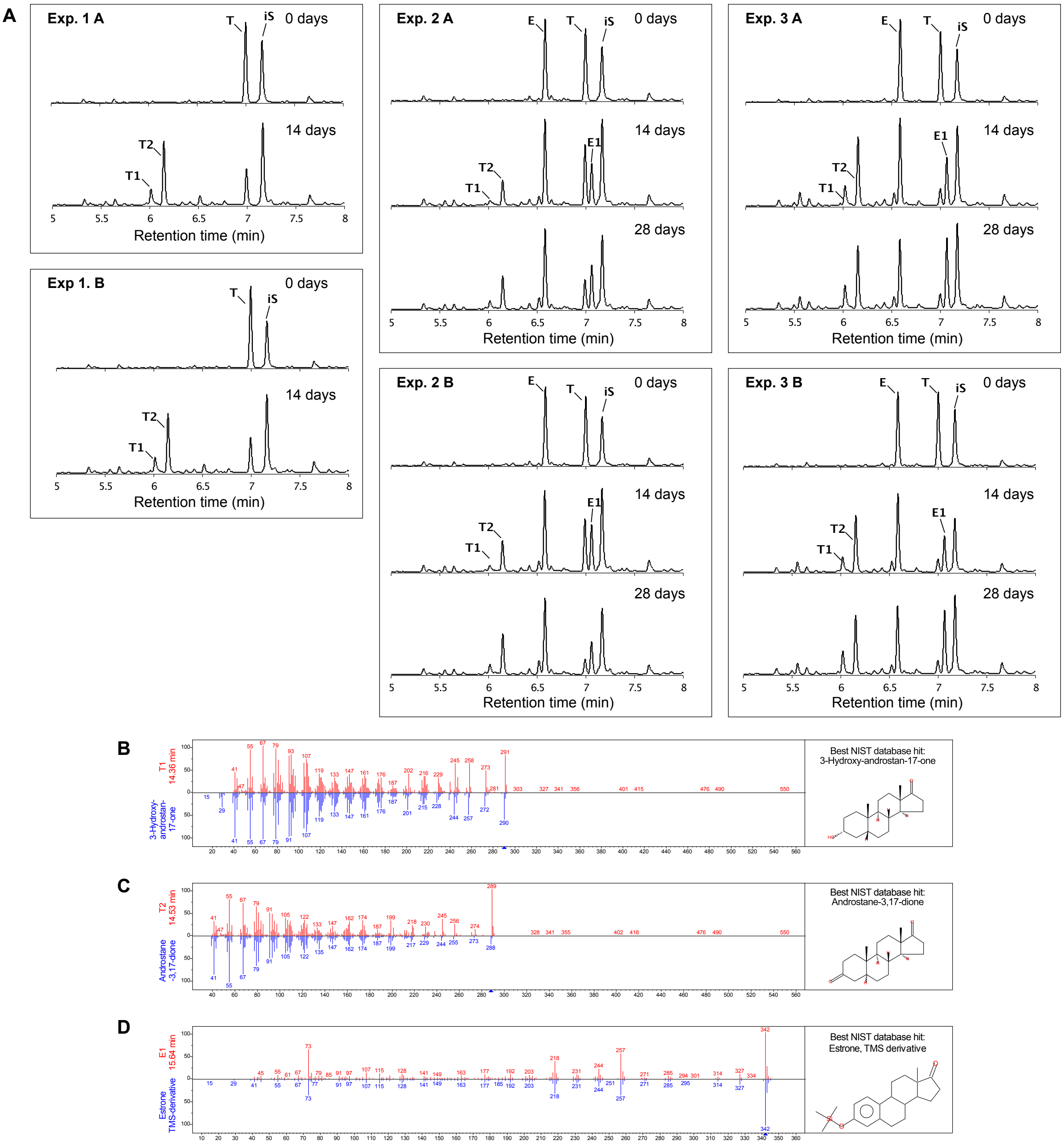

Supplemental figure S6: **(A)** GC-FID chromatograms of organic lipid extracts from methanogenic batch cultures from three independent experiments spiked with testosterone **(T)** and β-estradiol **(E)**. Whole cultures from duplicate setups (**A+B**) were harvested at the beginning of each experiment and after 14 and 28 days of incubation. Cholestane was used as internal extraction standard (**iS**). In all three experiments testosterone was transformed into two new products that were identified as **(B)** 3-hydroxy-androstan-17-one (**T1**) and **(C)** androstane-3-17-dione (**T2**) by GC-MS analysis and β-estradiol was transformed into one new product that was identified as **(D)** estrone (**E1**).

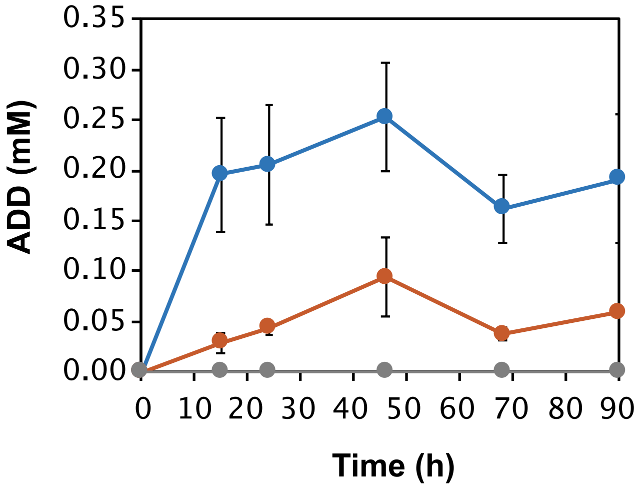

Supplemental figure S7: Formation of androstadienedione (ADD) in resting cell cultures of *Mycobacterium neoaurum* incubated with dried and homogenized solid digestate from biogas plant 1. In the presence of methyl-β-cyclodextrin a maximum of around 0.25 mM ADD was formed (blue line), while around 0.9 mM ADD were formed without methyl-β-cyclodextrin within 96 h (orange line). No ADD formation was observed in cultures without *M. neoaurum* (grey line).
